# Deep Learning Empowered Wearable-Based Behavior Recognition for Search and Rescue Dogs

**DOI:** 10.3390/s22030993

**Published:** 2022-01-27

**Authors:** Panagiotis Kasnesis, Vasileios Doulgerakis, Dimitris Uzunidis, Dimitris G. Kogias, Susana I. Funcia, Marta B. González, Christos Giannousis, Charalampos Z. Patrikakis

**Affiliations:** 1 Department of Electrical and Electronic Engineering, University of West Attica, 12244 Athens, Greece; v.doulger@uniwa.gr (V.D.); duzunidis@uniwa.gr (D.U.); dimikog@uniwa.gr (D.G.K.); giannousis@uniwa.gr (C.G.); bpatr@uniwa.gr (C.Z.P.); 2 Spanish School of Rescue and Detection with Dogs (ESDP), 28524 Madrid, Spain; s.izquierdo@escuelasalvamento.org (S.I.F.); esdp.eu@escuelasalvamento.org (M.B.G.)

**Keywords:** deep learning, canine activity recognition, bark detection, wearable computing, search and rescue system

## Abstract

Search and Rescue (SaR) dogs are important assets in the hands of first responders, as they have the ability to locate the victim even in cases where the vision and or the sound is limited, due to their inherent talents in olfactory and auditory senses. In this work, we propose a deep-learning-assisted implementation incorporating a wearable device, a base station, a mobile application, and a cloud-based infrastructure that can first monitor in real-time the activity, the audio signals, and the location of a SaR dog, and second, recognize and alert the rescuing team whenever the SaR dog spots a victim. For this purpose, we employed deep Convolutional Neural Networks (CNN) both for the activity recognition and the sound classification, which are trained using data from inertial sensors, such as 3-axial accelerometer and gyroscope and from the wearable’s microphone, respectively. The developed deep learning models were deployed on the wearable device, while the overall proposed implementation was validated in two discrete search and rescue scenarios, managing to successfully spot the victim (i.e., obtained F1-score more than 99%) and inform the rescue team in real-time for both scenarios.

## 1. Introduction

Animal Activity Recognition (AAR) and monitoring is an emerging research area enhanced mainly by the recent advances in computing, Deep Learning (DL) algorithms, and motion sensors. AAR attracted significant attention as it can provide significant insights about the behavior, health condition, and location of the observing animal [1]. In addition, if a proper network implementation is considered (e.g., with the proper devices, software, and communication protocol) the monitoring of the animal can be performed in real-time to allow exploitation of AAR for various purposes, e.g., study of the interaction between different animals, search and rescue missions [2], protection of animals from poaching and theft, etc. [3]. To perform this, the use of inertial sensors is mandated, such as accelerometers, gyroscopes, and magnetometers as well as a Machine Learning (ML) method, which after the proper training can accurately classify the animal activity [4].

Acknowledging the fact that AAR is a rich source of information that not only provides insights into animals life and well-being but also about their environment, over the past years, several works reporting on the use of animal activity recognition were published, increasingly focusing on the use of ML [5], while several open access datasets [6] were available, assisting the development of models and tools for accurate activity recognition of different animals.

In this work, we focus on the Dog Activity Recognition (DAR) for search and rescue (SaR) missions. SaR dogs are important assets in the hands of first responders due to their inherent talents with olfactory and auditory senses. However, in some cases the dog handler is impossible to be present in the same spot with the SaR dog, and thus, a life-critical amount of time is spent as the dog must return to the trainer and guide him to the victim [7]. To solve this problem, we introduce a novel implementation comprised of a wearable device, a base station, a cloud server, a mobile application, and Deep Convolutional Neural Networks (CNN), which were shown in [8,9,10] to be more accurate compared with that of other ML algorithms due to their ability to extract features automatically. More specifically, we developed a back-mounted wearable device for the SaR dogs that can:

1. collect audio and motion signals, exploiting its inertial sensors (e.g., 3-axial accelerometer and 3-axial gyroscope) and the embedded microphone;
2. process the produced sensor signals using DL algorithms (e.g., Deep CNNs);
3. communicate the critical message via the candidate network architecture; and
4. display in real-time the dog activity and location to its handler via a mobile application.

The proposed implementation is validated in two SaR scenarios managing to successfully locating the victim and communicating this message to the first responders in real-time with more than 99% F1-score.

In the rest of the paper, we analyze the related work in the field to provide a wider view in the problem we address (Section 2). In Section 3, we propose the core modules of our implementation along with their details and specifications, and we illustrate the overall network architecture developed to communicate the messages between the first responder and the SaR dog. Section 4 elaborates the data collection/annotation steps as well as the employed CNN architectures, while in Section 5 we evaluate the algorithmic results in terms of efficiency and efficacy. Next, in Section 6 the validation of the proposed solution is discussed, proving that our prototype satisfies all the desired functional and nonfunctional requirements. Finally, Section 7 discusses the obtained results, the limitations of the approach, and the future steps, while Section 8 concludes the paper.

## 2. Related Work

In the current section, we present the related works presenting results on canine behavior recognition, audio classification, and existing SaR systems based on animal wearables.

### 2.1. Activity Recognition

In prior research, animal activity recognition and monitoring was exploited to study various types of animals, spanning from livestock animals [10,11,12,13,14,15] to wild animals [16,17,18]. In the former case, the animal monitoring can (a) optimize the asset management, as the animals can be maintained always within preset “virtual fences”, (b) provide insights about the animals’ health through tracking the fluctuation on their activity levels, and (c) designate the optimal pastures. In wild animals, the animal activity monitoring can (a) minimize the poaching illegal activity and stock theft, (b) extract the state of health of the observed populations, and (c) assist the observations about the behavior of the wild animals and the interactions between them and other species.

In the category of pet animals, a literature review which analyzes the different technologies used to monitor various target features, such as location, health, behavior, etc. can be found in [19]. In the domain of DAR, these results can aid us towards a better interpretation of the everyday routine of the animals and their needs, which in turn can directly benefit the interaction with their handlers or can be exploited to perceive the behavior SaR units, providing valuable information to their trainers (e.g., victim discovery). The field of DAR emerged over the last decade due to the availability of low-cost sensors and smart devices that can acquire data and perform the ML algorithmic procedure in real-time [6,20,21,22,23,24,25,26,27,28]. Usually, the sensors are located in the back, collar, withers, and tail of the dog, while the employed sensors are mainly 3-axial accelerometers, 3-axial gyroscopes and sensors which monitor biometric data (e.g., heart rate). After completing the data collection process from the various sensors and performing their proper preprocessing, the data are then fed into an ML algorithm for training to classify any forthcoming activity.

For the purposes of DAR, various ML algorithms were utilized to attain sufficient accuracy. A k-NN classifier was employed in [21] to classify 17 different activities by studying the naturalistic behavior of 18 dogs attaining an accuracy of about 70%. In [25], the SVM (Support Vector Machines) classifier was applied into a dataset that comprised 24 dogs performing seven discrete activities and attained an accuracy of above 90%. Further, in [28], the accuracy of various ML classification algorithms was evaluated in a dataset comprising 10 dogs of different breeds, ages, sizes and gender performing seven different activities. The employed algorithms were Random Forest, SVM, k-NN, Naïve Bayes, and Artificial Neural Network (ANN). ANN outperformed the other four algorithms in activity detection, whilst Random Forest outperformed the other four in emotion detection. The attainable accuracy exceeded 96% in all cases. A recent study [6] in dog behavior recognition examined the optimal sensor placement in the dog, through a comparison of various algorithms (e.g., SVM). In particular, the authors attached two sensor devices to each dog, one on the back of the dog in a harness and one on the neck collar. The movement sensor at the back yielded up to 91% accuracy in classifying the dog activities and the sensor placed at the collar yielded 75% accuracy at best. These results helped the current work to decide the optimal sensor placement, which was mounting a harness on the back of the SaR dog with the developed device in it. Finally, the authors in [29] created a huge dataset exploiting a 3-axial accelerometer and collecting data from more than 2500 dogs of multiple breeds. Then they trained a deep learning classifier which was then validated for a real-world detection of eating and drinking behavior. The validated results attained a true positive rate of 95.3% and 94.9%for eating and drinking activities, respectively. The details of the related work on DAR are shown in Table 1.

### 2.2. Audio Classification

Similar to wearable-based activity recognition, over the last years, there were proposed several audio signal processing techniques relying on DL algorithms and were proved to achieve better results than baseline ML algorithms [30]. DL algorithms, such as Deep CNNs, possess the ability to increase their performance as the training dataset grows; thus, the authors in [31] applied well-known CNN architectures, which were employed successfully in computer vision tasks, to test their effectiveness on classifying large-scale audio data. The networks architectures they used were a fully connected ANN, an AlexNet [32], a VGG [33], an Inception V3 [34], and a ResNet-50 [35]; these networks were trained and evaluated using AudioSet, which consists of 2,084,320 human-labeled 10-second audio clips drawn from YouTube videos. The audio classes are based on an audio ontology [36], which is specified as a hierarchical graph of event categories, covering a wide range of human and animal sounds, musical instruments and genres, and common everyday environmental sounds. Their experiments showed that the ResNet-50 model, which had the most layers (i.e., it was deeper than the others), achieved the best results.

In addition to this, CNNs are also state-of-the-art models, even for relative smaller audio datasets consisting of a few thousand samples. Salamon and Bello [37] compare a baseline system (i.e., using MFCCs features) with unsupervised feature learning performed on patches of PCA-whitened log-scaled mel-spectrograms using the UrbanSound8K dataset. In particular, they utilized the spherical k-means algorithm [38] followed by the Random Forests algorithm and managed an average classification accuracy 5% higher than the baseline system. Furthermore, Karol J. Piczak [30] obtained state-of-the-art results for the UrbanSound8K dataset, training a relatively shallow CNN (two convolutional layers), which had as input the log-scaled mel-spectograms of the audio clips. The proposed CNN model had an average accuracy of about 73.1% against the 68% average accuracy of the baseline model, despite the fact that it seemed to overfit the training data. A deeper, VGG-like CNN model (five convolutional layers) was implemented by A. Kumar [39] and used on the UltraSound8K dataset, reaching a 73.7% average accuracy.

Finally, data augmentation techniques were adopted by the researchers to increase the number of the audio samples. To this end, Salamon and Bello [40] explored the influence of different augmentation techniques ((a) Time Stretching; (b) Dynamic Range Compression; (c) Pitch Shifting; and (d) Adding Background Noise) on the performance of a proposed CNN architecture, and they obtained an average accuracy close to 79% using recordings of the ESC-50 (2000 clips) and ESC-10 (400 clips) datasets [41]. Moreover, Karol J Piczak [41] utilized random time delays to the original recordings of the ESC-50 and ESC-10 datasets. The CNN architecture achieved better accuracy results from the baseline model for both datasets, while in the case of the ESC-50, the difference between the average accuracies was over 20% (baseline accuracy: 44%, best CNN: 64.5%).

### 2.3. Existing SaR Solutions Based on Animal Wearables

SaR systems are vital components when it comes to disaster recovery due to the fact that every second might be life-critical. Trained animals, such as dogs (i.e., K9s), are exploited by SaR teams due to their augmented senses (e.g., smell), and their small size is ideal for searching under the debris for survivors.

The authors in [2] developed a two-part system consisting of a wearable computer interface for working SaR dogs communicating with their handler via a mobile application. The wearable comprised a bite sensor and a GPS to display the K9s location in the mobile application. The SaR dog bites the bringsel, which is equipped with the bite sensor to notify its handler. In addition to this, the work in [42] demonstrates several interfaces developed for animal–computer interaction purposes, which could be used in SaR missions for notifying the canine handler, such as bite sensors, proximity sensor, and tug sensor. Furthermore, in [7,43] the use of head gestures is examined to establish communication between the SaR dogs and the handlers. The developed wearable is added in a collar and is comprised by motion sensors (3-axial accelerometer, gyroscope and magnetometer), while the systems analyzes motion signals produced by the canine wearable using dynamic time warping. Each detected head gesture is paired with a predetermined message that is voiced to the humans by a smart phone. To this end, the participating K9s were specifically trained to perform the appropriate gesture.

Existing patented canine wearables, such as [44,45], could also be used for SaR purposes. A wirelessly interactive dog collar is presented in [45]; it allows voice commands and tracking over long distances, along with features that facilitate tracking and visualization, exploiting its embedded sensors (GPS, microphone, speaker, light). Moreover, in [44] an enhanced animal collar is presented. This device consists of extra sensors, such as camera, thermographic camera, and infrared camera to enable the transmission of the captured images, in addition to audio signals.

Finally, the animal-machine collaboration was also explored. The authors in [46] introduce a new approach to overcome the mobility problem of canines through narrow paths in the debris utilizing a robot snake. The SaR dog carries this small robot, and when it is close to the victim it barks to release the robot that locates the trapped person. The robot snake is equipped with a microphone and a camera. Rat cyborg is another option for SaR missions [47]. The system is implanted with microelectrodes in the brain of a rat, through which the outer electrical stimuli can be delivered into the brain in vivo to control its behaviors. The authors state that the cyborg system could be useful in search and rescue missions where the rat handler can navigate through the debris by exploiting a camera mounted on the rats.

Table 2 summarizes the aforementioned works including our solution, in terms of equipped sensors/actuators and their capabilities (i.e., communication with handler and victim, edge data processing, delivery package, search through debris, extra animal training, no welfare concerns, no rescuer guidance needed).

## 3. Network Architecture

**Figure 1:**
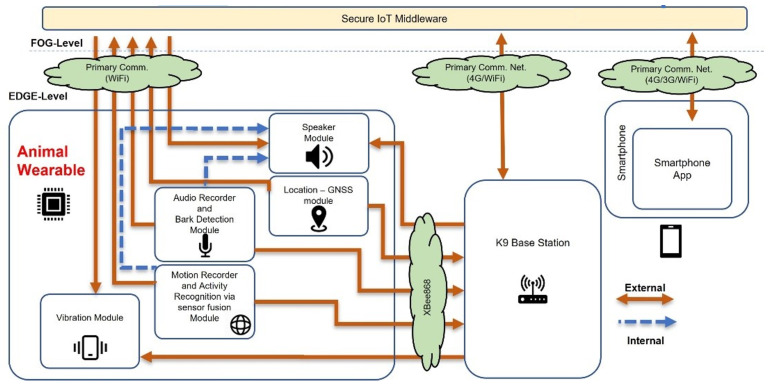
Wearable for animals—system architecture and communication flows.

The overall system architecture for the SaR dog real-time monitoring is illustrated in Figure 1. The architecture is divided into two levels (i.e., layers):

1. the EDGE-level, which contains the architectural modules that have low computational power and are located at the edge of the network (i.e., wearable device, base station and the smartphone application), and
2. the FOG-level, which contains the modules having higher computational power and enhanced communication capabilities (publish-subscribe middleware and the local Portable Command Center (PCOP), used during a SaR mission).

The communication from the EDGE layer to the FOG takes place mainly between the wearable device and the Secure IoT Middleware (SIM), which contains an encrypted KAFKA (https://kafka.apache.org/, accessed on 22 September 2021) Publish-Subscribe broker using Wi-Fi connectivity. When such connectivity is not available at the area of operation, a secondary communication path is deployed. This path represents communication at the EDGE layer and includes an RF (Radio Frequency) connection between the wearable and the Base Station (BS). Once the data are collected by the BS, it uses a Wi-Fi/3G/4G connection to publish them at the FOG layer’s KAFKA pub-sub broker through the SIM.

Finally, the smartphone of the first responder, which is the handler of the animal, is notified in real-time about the SaR dog’s behavior by the KAFKA broker via his/her mobile application. All these data flows can be seen in Figure 1, while the developed EDGE-level modules are explained in detail in the following subsections.

### 3.1. Wearable Device

The wearable device is the most important module as it collects various types of data, such as 3-axial accelerometer and 3-axial gyroscope data, audio recordings, and localization data, while it can also provide feedback to the dog via vibration and audio signals. The wearable was developed from our team with the guidance of K9-SaR experts solely for the purposes of the SaR task; however, it can be exploited for various other tasks comprising animal activity monitoring and recognition, e.g., in dogs or even in livestock animals for the purposes of behavioral analysis.

The designed harness is back-mounted vest instead of neck-mounted (i.e., collar) to further improve the animal’s comfort by moving the center of mass to a more suitable place, as well as to achieve higher accuracy for the activity recognition task [6]. The new design is completely modular since all the components are attached with Velcro to the wearable. A strip with Velcro is also included at the belly of the animal to provide for further grip. In general, the detachability requirement is related to the dog’s safety, as it ensures that the dog will break free from the wearable if tangled.

**Figure 2:**
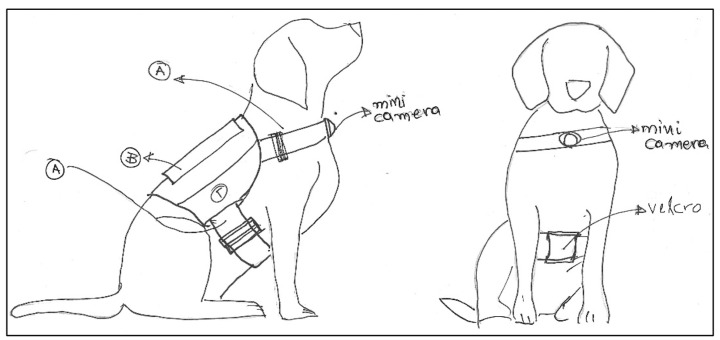
Drawing displaying placement of animal wearable.

In Figure 2 the sketch for the designs of the animal wearable is displayed including the strips with velcro attachments (points *A* and Γ), the pouch for electronics (point *B*) and the mini-camera position at the animal’s front. In particular, point *B* (back of the animal) contains the main computational platform, the custom board and the battery, while the camera is placed on the animal’s chest, always facing in front.

**Figure 3:**
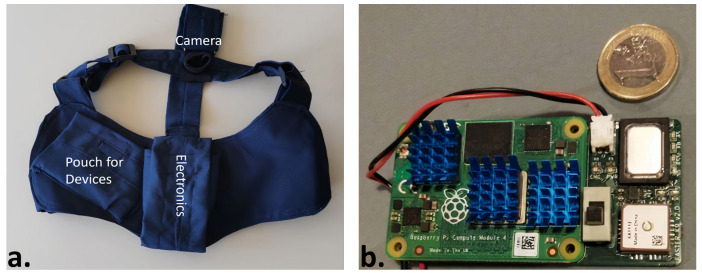
Designed animal harness (**a**) and electronic device (**b**).

Additionally, except from the pouch for the electronics, another, optional, smaller pouch/pocket was introduced, able to fit a small device (e.g., a mobile phone), or any small item considered useful to be carried by the animal (Figure 3a). This design pertains to rescue scenarios where the delivery of a small item to an unreachable trapped person could be of great importance and contribute to the efficient rescue.

The main features of the wearable device, which is pictorially described in Figure 3b, are the:

- Hardware (processing unit): Raspberry Pi 4 Compute Module (https://www.raspberrypi.com/products/compute-module-4/, accessed on 16 December 2022) (CM4), which features the Broadcom BCM2711, a quad-core ARM Cortex-A72 processor chosen as a processor for the SaR wearable, with dimensions of about 55 × 40 mm. The CM4 offers the processing power required for the complete list of features of the device, including the inference of the DL models as well as the hardware peripherals needed to drive the sensors and modules of the custom board.
- Power Supply Unit: Texas Instruments TPS63002, a Single Inductor Buck-Boost Converter. Since the Raspberry Pi requires a 5 V power supply, a Buck-Boost Converter offers the ability to use a single cell 3.6 V battery and still provide the desired 5 V to the device. The specific model was selected due to its QFN package, measuring 3 mm × 3 mm and due to the small number of additional components needed for the power supply, keeping the overall size on the board minimal.
- Battery Charger Module: Texas Instruments BQ24075 standalone 1-Cell Li-Ion 1.5-A Linear Battery Charger. This battery charger, being in a QFN package offers a small footprint on the device of only 3 mm × 3 mm, and the simplicity of the application circuit assists to the minimalization of the device dimensions. The maximum battery charging current was set to 1.0 A offering a balance between a quick charge for most commercially available batteries and a battery health preservation by keeping the charging rate under 0.5C.
- Battery: 18650 Li-Ion battery cell. The device was designed to operate with a single 18650 type cell, due to the wide adoption of this specific rechargeable battery format, which helps lower the cost of the device and allows for easier maintenance, while offering a balanced solution between energy capacity, size and weight. The operation duration after which the battery was selected is 1 h, which was met with a cell of 3000 mAh. Longer operations can still be covered by carrying multiple spare batteries, since it is a battery type that can be easily replaced in the field.
- RF module: XBee SX 868 modules manufactured by Digi, offering a maximum transmission current of 55 mA. The communication data rate between two modules is set to 10 Kbps to maximize the range and the module is connected to the main processor through the UART interface. These radio modules claim a theoretical maximum range of 14.5 km in line-of-sight with a 2.1 dBi antenna and a maximum transmission current of 55 mA. A maximum range of 750 m was achieved in a line-of-site urban environment. They were chosen over the competition with similar performance, for their setup simplicity and the ability to form a network with as low as two identical modules without the need of third-party involvement and/or subscription fees.
- Audio recording module: SPH0645LM4H-B MEMS digital microphone by Knowles. A very small low-power omnidirectional digital microphone was needed, which uses the I^2^S audio bus and requires very few additional components, keeping the device’s dimensions to a minimum.
- Audio playback module: the audio signal is constructed from the PWM output of the processor and then it is amplified through Texas Instruments’ TPA711DGNR19 low-voltage audio power amplifier, a mono amplifier capable of conducting up to 750 mW of RMS power to an 8Ω speaker continuously. The amplifier’s footprint measures 5 mm × 3.1 mm, although the complete audio circuitry also includes an electrolytic capacitor measuring 4.3 mm × 4.3 mm × 5.5 mm.
- Inertia Measurement Unit: BMI160 from Bosch Sensortec featuring a 16-bit triaxial accelerometer and a 16-bit triaxial gyroscope. The module is selected for its very low power consumption of 925 μ A and its very small footprint of 2.5 mm × 3 mm. Both the accelerometer and the gyroscope can be operated at high sampling rates of 3200 Hz and 800 Hz respectively, well above the 100 Hz needed for this project.
- GNSS module: Sierra Wireless XA1110 offering a mix of GPS, GLONASS and Galileo satellite system tracking with a maximum update rate of 10 Hz with an integrated patch antenna. This module was selected for its small size of 12.5 × 12.5 × 6.8 mm, which was preferable than a possibly smaller module combined with a larger separate antenna requiring additional space.
- Vibrator module: Texas Instruments’ DRV2603 Haptic Drive. The haptic drive comes in a QFN package measuring only 2 mm × 2 mm contributing to the small size needs of the device.
- Camera module: The Raspberry Pi Camera Module v1 was initially selected, but was later dropped in favor of a standalone mini actioncam like the SQ12. This change allowed for a modular design, where the camera can be used when needed or removed when not.

The custom board was designed to meet or exceed the predefined specifications covering device functionality and achieve a balance between battery life, weight, and physical dimensions. Therefore, in most cases, the chosen modules are the smallest that would satisfy the consumption and functional requirements. The total weight of the device is 121 g including the battery (47 g), while the total cost for ordering the components and assembling them was equal to 260€. Table 3 provides details on the electrical characteristics, maximum ratings and recommended operating conditions of the device.

### 3.2. SaR Base Station

The BS device is a portable wireless device, based on the Raspberry Pi Zero W (https://www.raspberrypi.com/products/raspberry-pi-zero-w/, accessed on 19 January 2022) and powered by an internal power-bank. It is equipped with an XBee SX 868 RF module (https://www.digi.com/xbee, accessed on 19 January 2022) similar to the wearable devices and creates an XBee network to which all animal wearable devices in range can connect. This results in an extended range of coverage for the animal wearables. The BS device, includes a pocket Wi-Fi module, granting 4G connectivity. Any messages sent from the wearables are received by the BS through the Xbee network and delegated to the SIM over either the Wi-Fi connection to the Pocket Wi-Fi device and then transmitted over 4G network, or to any other known Wi-Fi hot spot. Likewise, any commands issued by the rest of the modules to the wearables (e.g., initiate data collection), are either received directly by the devices through Wi-Fi, or received by the BS and relayed to the devices via the XBee network. The existence of a BS is extremely critical in a disaster scenario, as public telecommunications networks cannot be taken for granted. For this purpose, the animal wearable device cannot rely solely on mobile network coverage.

### 3.3. Smartphone Application

The application of the animal wearable is one feature of a wider application developed for FASTER (First responder Advanced technologies for Safe and efficienT Emergency Response) EU Horizon 2020 project (https://cordis.europa.eu/project/id/833507, accessed on 16 December 2021). As a result, the application contains four tabs for displaying: (a) biometrics of first responders, (b) environmental data, (c) the behavior and location of SaR dogs, and (d) upcoming notifications (e.g., a victim was found). In general, it is an Android application (supporting android version 8.0 and above) that makes use of Google Maps (https://www.google.com/maps, accessed on 16 December 2021) for the depiction of information about the location of the dog. The application receives the information from a KAFKA broker with the aid of a Quarkus Reactive Streams (https://quarkus.io/, accessed on 16 December 2021) service. The information flows continuously from KAFKA to the screen of the user. Reactive streams work by publishing messages whenever they receive new information from a source. This makes the information flow “seamless” and most importantly it does not spam the server with http requests every some seconds. The android system can absorb these streams with the use of a library called okSse (https://github.com/biowink/oksse, accessed on 16 December 2021) which helps to establish a connection with a reactive streams service.

**Figure 4:**
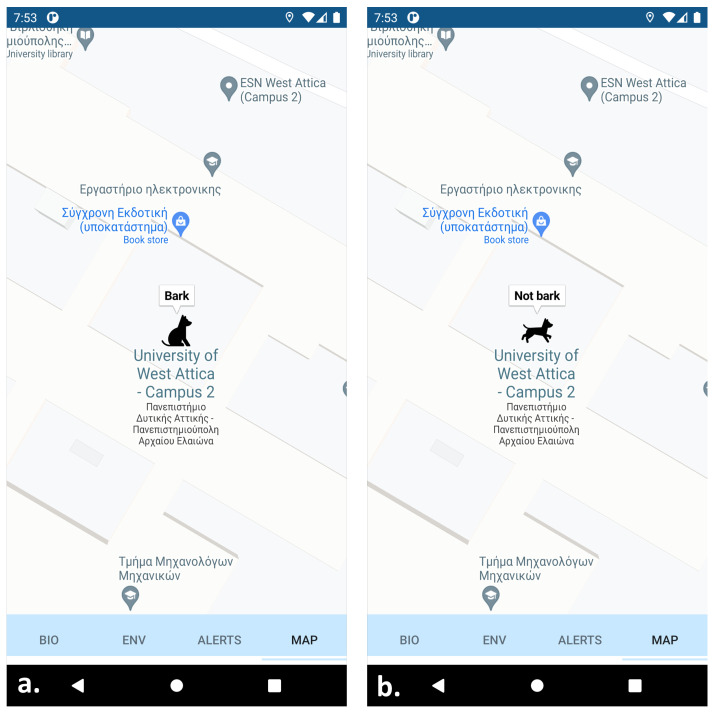
Developed smartphone application displaying SaR dog behavior: (**a**) canine is not moving and barks; (**b**) canine is moving and does not bark.

Once we get the information, we feed it to our system with the use of LiveData (https://developer.android.com/topic/libraries/architecture/livedata, accessed on 16 December 2021). LiveData is an observable data holder class. Unlike a regular observable, LiveData is lifecycle-aware, meaning it respects the lifecycle of other application components, such as activities, fragments, or services. This awareness ensures LiveData only updates the application component observers that are in an active lifecycle state. With the use of an observer, we “observe” any changes to the state of the information, and when we find something new we draw on the map the new location or behavior of the dog. The dog actions describe the state in which the animal is at a particular time in space (Figure 4). For example, whether the dog is walking/running or standing still.

## 4. Data Collection, Processing, and Deployment of Deep Learning Algorithms

### 4.1. Data Collection Process

**Figure 5:**
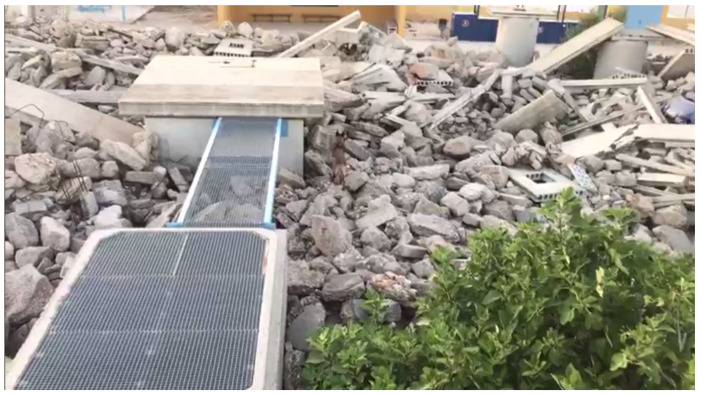
Arena used to conduct search and rescue tests.

The tests were performed in an arena covered with ruins to mimic a real search and rescue operation as best as possible (Figure 5). The tests included search and rescue missions both during the day and night. In the former case, adequate vision is considered, while in the latter, only limited vision can be attained. The resulting AI algorithms are trained in both cases, as in a real operation both cases can be encountered.

Next, the testing procedure is as follows. First, a member of the rescuing team, the “victim”, hides somewhere in the arena among the ruins, in one of the various spots which are designed for this purpose. Then, after the wearable on the SaR dog is activated by his trainer, the dog is allowed to search for the victim. The test is successfully completed when the SaR dog is able to found the “victim”. In this successful case, the SaR dog makes a characteristic bark sound, which lasts for some seconds, while it is in a standing position and stares at the “victim”. Depending on the location of the “victim” in the arena, the search and rescue test may last from half a minute up to a few minutes.

### 4.2. Labeling Process

The labeling process was performed offline using video and audio recordings. The videos were recorded using a smartphone camera which was positioned on a high place on one side of the arena to capture almost the entire search and rescue field. The audio recordings were performed using the wearable device’s microphone. Only segments longer than 2 s were considered during the labeling process, which means that a single activity needs to last more than two consecutive seconds to be labeled. The recorded videos were synchronized with sensor data using metadata (e.g., timestamp) and via exploiting the plotted time series of the sensors (e.g., accelerometer). Four activities were considered:

(a) standing—the dog is standing still on four legs without its torso touching the ground, occurring mainly when the dog successfully finds the victim;
(b) walking—the dog moves at slow speed and its legs are moving one after another;
(c) trotting—the dog moves at a faster speed than walking and slower than running. This is the most frequent movement activity during the search and rescue operation; and
(d) running—the dog moves at a very fast speed, occurring mainly when the dog is released by its trainer at the beginning of the search and rescue operation.

**Figure 6:**
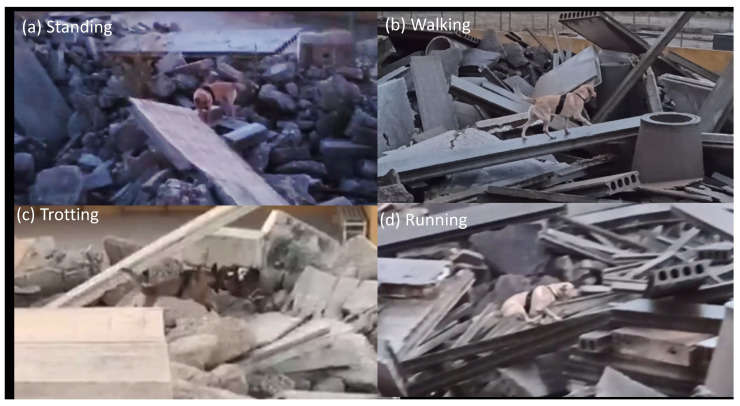
Instances of four activitie.

In cases where it was not possible to identify the dog activity, either due to insufficient light during the night operation or when the dog was not clearly shown in camera, (e.g., it was behind an obstacle) a “missing” label was considered. These data were omitted for the Artificial Intelligence (AI) training procedure. Next, the audio recordings include only two classes, barking and nonbarking, as the barking is the required state that designates that the SaR dog spotted the “victim”. Examples of the four dog activities are shown in Figure 6.

### 4.3. Details of the Created Dataset

**Figure 7:**
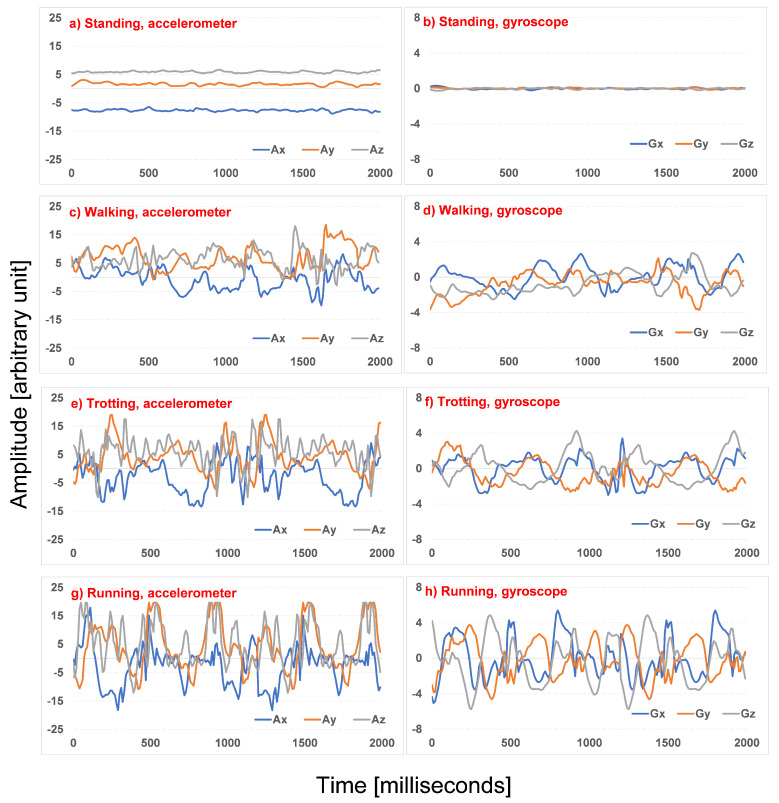
Sensor samples of 2 s duration for four activities.

The complete dataset comprises nine dog search and rescue sessions. After the labeling process, each session is segregated in various segments, where each segment comprises only one activity, considering a minimum segment duration of 2 s. Each second of raw data consists of 100 values for the two 3-axial sensors (3-axial accelerometer and 3-axial gyroscope) forming a total of 600 values. Next, each segment is segregated in samples with a 2 s length where a 50% overlap is considered. An example of samples for the four SaR dog activities from both the accelerometer and the gyroscope is illustrated in Figure 7. Evidently, the amplitude of the accelerometer and the gyroscope increase as the activity becomes more intense, which means that the lowest amplitude can be found in standing and the highest in running.

Further, the dataset details for all seven search and rescue testing sessions are tabulated in Table 4. Evidently, the most frequent activities are standing and trotting. This is expected, as during the search and rescue operation, on one hand the dog trots while searching for the “victim” between the ruins and on the other hand, when the “victim” is found, the dog remains in a standing position and barks. Moreover, only one of the K9s provided a sufficient amount of “running” examples (session 4), and only two canines sufficient amount of “standing” examples (session 4 and 6). Thus, by adopting a leave one subject out approach, it is impossible to check the model’s generalizability on the classes “running” and “walking”, and, as a result, we merged the motion activities “running”, “walking” and “trotting” into one class, called “searching”.

Turning our attention to the bark detection, similar to SaR dog activity detection, the labeling process was performed offline using the provided audio recordings and it was compared with the video recordings to verify the annotations. The annotated data were afterwards segmented into 2 s audio clips. This window size was selected to reduce the throughput to the developed model.

Another reason for selecting 2 s was to match the window size of the Inertial Measurement Unit (IMU) data and, also, to have a better understanding of the situation the SaR dog is into. For example, in the case of real-time inference and for e.g., a 4 s window, if the dog barks in the first second of the audio stream the model would still classify it as bark, despite the fact this occurred 3 s ago.

The dataset we built consists of 1761 examples (i.e., audio clips), where 258 are audio clips containing bark and 1503 do not, leading to an unbalanced dataset, which however reflects a real-world search and rescue operation. Before introducing the data in the Deep CNN, we split them into three subsets, namely training set, validation set, and test set, following the standard procedure of training an neural network. The train set contains around 74% of the data, the validation set around 10% of the data and the test set around 16% of the data. The split was performed based on the search and rescue sessions. i.e., audio signals recorded during a specific search and rescue session belong to the same dataset, avoiding in this way overlapping samples between the different sets or characteristic bark patterns.

### 4.4. Developed DL Algorithms

#### 4.4.1. Activity Recognition

**Figure 8:**
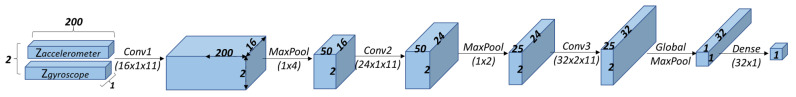
Overall architecture of developed Deep CNN for activity recognition task. Input tensor has two rows representing produced Z(i) for accelerometer and gyroscope, each one of them containing 200 values and one channel. Every convolutional operation is followed by a ReLU activation function, and pooling layers are followed by a dropout equal to 0.5. Final dense layer outputs one value and is followed by a sigmoid operation that represents probability of SaR dog searching or standing.

The employed Deep CNN for the dog activity recognition is a lightweight architecture to be deployed on the animal wearable (i.e., contains around 21,400 parameters), it is based on late sensor fusion [8] (i.e., the fist convolutional layers process the input signals individually) and consists of the following layers (Figure 8):

- layer 1: sixteen convolutional filters with a size of (1, 11), i.e., $W_{1}$ has shape (1, 11, 1, 16).
  This is followed by a ReLU activation function, a (1, 4) strided max-pooling operation and a dropout probability equal to 0.5.
- layer 2: twenty-four convolutional filters with a size of (1, 11), i.e., $W_{2}$ has shape (1, 11, 16, 24).
  Similar to the first layer, this is followed by a ReLU activation function, a (1,2) strided max-pooling operation and a dropout probability equal to 0.5.
- layer 3: thirty-two convolutional filters with a size of (2, 11), i.e., $W_{3}$ has shape (2, 11, 24, 32).

The 2D convolution operation is followed by a ReLU activation function, a 2D global max-pooling operation and a dropout probability equal to 0.5.

- layer 4: thirty-two hidden units, i.e., $W_{4}$ has shape (32, 1), followed by a sigmoid activation function.

Before feeding the algorithms with the collected data, we performed a preprocessing routine as follows. To acquire orientation independent features, we calculated a 3D vector (the l2-norm) from the sensors’ individual axes [48]. The orientation-independent magnitude of the 3D-vector is defined as:

(1) $$S\left(i\right)=\sqrt{s_{x}^{2}\left(i\right)+s_{y}^{2}\left(i\right)+s_{z}^{2}\left(i\right)}$$

where $s_{x}\left(i\right)$, $s_{y}\left(i\right)$, and $s_{z}\left(i\right)$ are the three respective axes of each sensor (accelerometer and gyroscope) for the *i*^th^ sample. Then, the dataset is divided seven-fold (i.e., one per session). To obtain subject independent results and evaluate the generalization of the algorithms, we used five folds as a training set, one as a validation set, and one as a test set. Afterwards, a circular rotation between training, validation and test subsets was performed to ensure that the data from all sessions will be tested. Finally, each sensor’s values (obtained by Equation (1)) were normalized by subtracting the mean value and dividing by the standard deviation (calculated by the examples included only in the training set), defined as:

(2) $$Z\left(i\right)=\frac{S(i)-\mu }{\sigma }$$

where S(i) denotes the *i*^th^ sample of a particular sensor (e.g., accelerometer), Z(i) its normalized representation and μ and σ denote their mean and standard deviation values, respectively.

#### 4.4.2. Bark Detection

**Figure 9:**
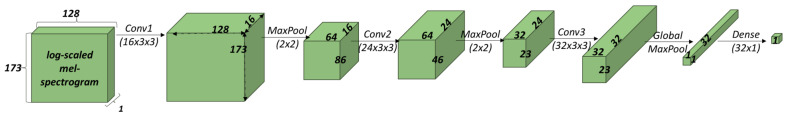
Overall architecture of developed Deep CNN for bark detection task. Input tensor is log-scaled mel-spectrogram, with 173 rows, each one of them containing 128 values (mels) and one channel. Every convolutional operation is followed by a ReLU activation function, and pooling layers are followed by a dropout equal to 0.5. Final dense layer outputs one value and is followed by a sigmoid operation that represents probability of SaR dog barking or not barking.

For the task of bark detection, we evaluated two different strategies. The first one is based on a large pretrained model where we applied transfer learning, i.e., we finetuned its weights using the dataset we collected. In particular, we selected the model introduced in [49] that achieved state-of-the-art results in the ESC dataset [41]. The code for reproducing the model is publicly available (https://github.com/anuragkr90/weak_feature_extractor, accessed on 12 September 2021). The latter was a custom lightweight (i.e., contains 10,617 parameters) Deep CNN architecture and consists of the following layers (Figure 9):

- layer 1: sixteen convolutional filters (i.e., kernels) with a size of (3, 3), i.e., $W_{1}$ has shape (3, 3, 1, 16)

This is followed by the ReLU activation function, a strided (2, 2) max-pooling operation and a dropout probability equal to 0.5.

- layer 2: twenty-four convolutional filters with a size of (3, 3), i.e., $W_{2}$ has shape (3, 3, 16, 24).

Similar to the first layer, this is followed by a ReLU activation function, a (2,2) strided max-pooling operation and a dropout probability equal to 0.5.

- layer 3: thirty-two convolutional filters with a size of (3, 3), i.e., $W_{3}$ has shape (3, 3, 24, 32).

The 2D convolution operation is followed by a ReLU activation function, a global max-pooling operation, and a dropout probability equal to 0.5.

- layer 4: thirty-two hidden units, i.e., $W_{4}$ has shape (32, 1), followed by a sigmoid activation function.

Before injecting the collected audio data in the CNN, we performed data normalization by dividing all the values with the max value included in the sample. Afterwards, the log-scaled mel-spectrograms were extracted from the audio clips having a window size of 1024, hop length of 512 and 128 mel-bands. Moreover, the segments of each clip overlapped 50% with the previous and the next one, and we discarded a lot of silent segments since they increased significantly the number of not-bark examples without, however, increasing the model’s performance.

**Figure 10:**
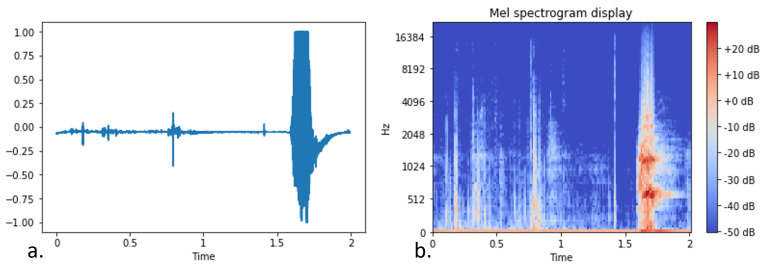
Raw representation (**a**) and log-scaled mel-spectrogram (**b**) of a dog barking audio signal.

**Figure 11:**
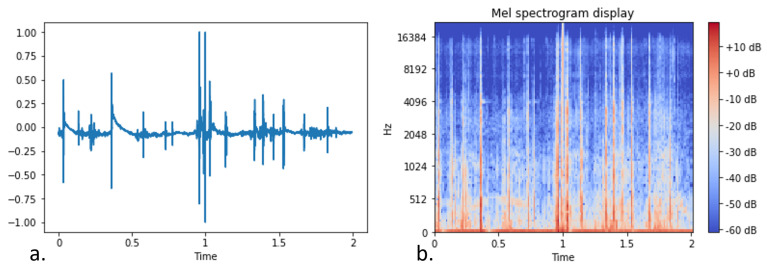
Raw representation (**a**) and log-scaled mel-spectrogram (**b**) of a dog not barking audio signal (dog running).

Figure 10 and Figure 11 visualize the transformation of a clip containing bark and a clip including nonbarking activity, respectively. The comparative difference between the barking and the nonbarking state is obvious both in the raw data representation and in the mel-spectrogram.

## 5. Results

### 5.1. Results on the Activity Recognition

In this section, we benchmark the proposed CNN against four other machine learning algorithms, namely Logistic Regression (LR), k-Nearest Neighbours (k-NN), Decision Tree (DT), and Random Forest (RF). For these algorithms we opted to extract the same seven time-dependent features for each sensor (accelerometer and gyroscope), resulting in 14 features in total (see Table 5). The ML experiments were executed on a computer workstation equipped with an NVIDIA GTX 1080Ti GPU, which has 11 gigabytes RAM, 3584 CUDA cores, and a bandwidth of 484 GB/s. Python was used as the programming language, and specifically the Numpy for matrix multiplications, data preprocessing, segmentation, and transformation and the Keras high-level neural networks library using as a backend the Tensorflow library. To accelerate the tensor multiplications, CUDA Toolkit in support with the cuDNN was used, which is the NVIDIA GPU-accelerated library for deep neural networks. The software is installed on a 16.04 Ubuntu Linux operating system.

The proposed CNN model was trained using the Adam optimizer [50] with the following hyper-parameters: learning rate = 0.001, $beta_{1}$ = 0.9, $beta_{2}$ = 0.999, epsilon = $10^{-8}$, decay = 0.0. Moreover, we set the minimum number of epochs to 500; however, the training procedure terminated automatically whether the best training accuracy improved or not after a threshold of 100 epochs. The training epoch that achieved the lowest error rate on the validation set was saved, and its filters were used to obtain the accuracy of the model on the test set.

Table 6 presents the accuracy results that were obtained on applying the aforementioned algorithms and the developed Deep CNN architecture on the SaR dog activity recognition dataset. The presented results were obtained per dog having different folds in the test set (i.e., 5-fold cross-validation), while we made five runs for each to avoid reducing the dependency on different weights initializations and averaged them afterwards. The highest accuracy was achieved by the Deep CNN model (93.68%), which surpassed importantly the baseline algorithms, especially DT and k-NN. Moreover, having the algorithms achieved the best results (98.57% averaged accuracy) having dog five in the test set and the worst ones when they were evaluated on the dog seven examples (83.57% averaged accuracy). In addition to this, through the following table we can observe that k-NN had the biggest deviation in terms of accuracy among the seven subjects (i.e., dogs) ranging from 73.34% to 100%, while the RF was the smallest one, ranging from 84.34% to 100%.

**Figure 12:**
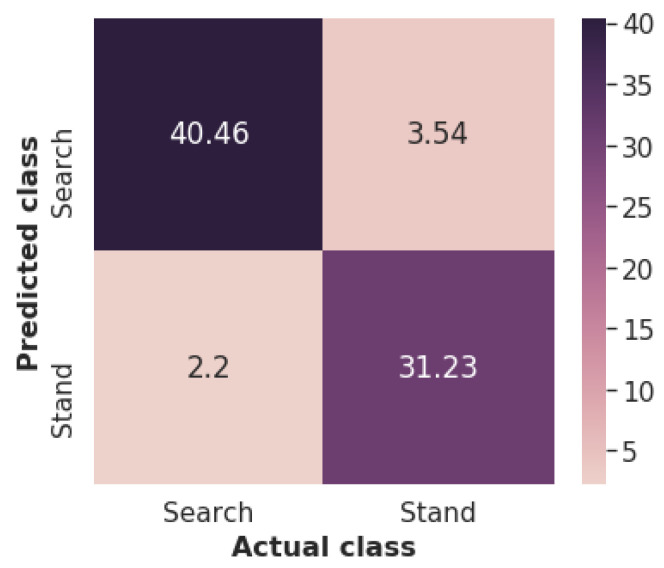
Confusion matrix (averaged over different test sets) of developed deep CNN.

Figure 12 displays the confusion matrix of the developed deep CNN averaged over the different test sets. The false positives (i.e., examples falsely predicted as “stand”) are more than the false negatives (i.e., examples falsely predicted as “search”), which is somewhat unexpected since the “search” class contains more examples than the “stand” class. However, after performing error analysis on the obtained results we noticed that 11 out of the 65 walking activities, were falsely classified as “stand”. This misclassification concerning the SaR dogs’ low intense activities adds around 1.52 false positives, and without it, the portion of false-positive and negatives would be almost equal.

### 5.2. Results on the Bark Detection

We followed the same experimental set-up that described in section for activity recognition regarding the workstation used, the libraries, and the optimizer. The hyperparameters of the Deep CNN were: learning rate = 0.001, $beta_{1}$ = 0.9, $beta_{2}$ = 0.999, epsilon = $10^{-8}$, decay = 0.0, while Adam optimizer is also considered. Moreover, we set the minimum number of epochs to 1000; however, the training procedure terminated automatically whether the best training accuracy had improved or not, after a threshold of 100 epochs. Similar with the case of the CNN in the activity recognition, the training epoch that achieved the lowest error rate on the validation set was saved, and its filters were used to obtain the accuracy of the model on the test set.

The results on the test set of the developed search and rescue dataset are presented in Table 7 the best results were achieved exploiting the Deep CNN after applying transfer learning using the Deep CNN in [49] (named as Deep CNN TL). The attainable accuracy of our model is 99.13% and the F1-score is 98.41%, while the Deep CNN TL achieved 99.34% accuracy and 98.73% F1-score.

**Figure 13:**
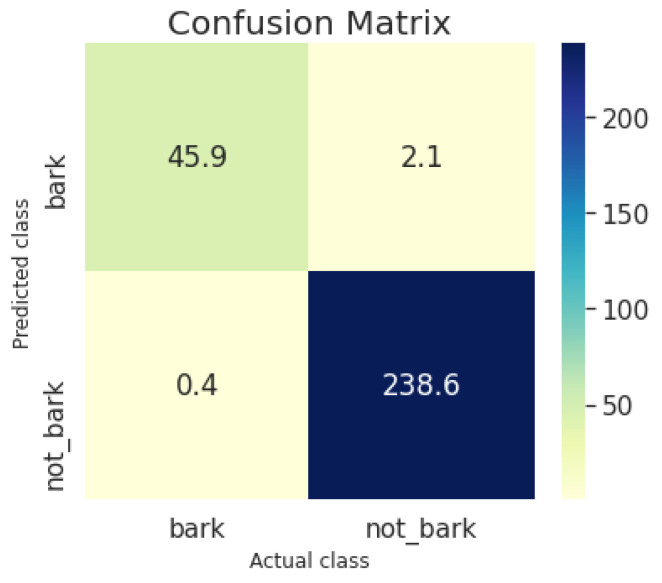
Confusion matrix of Deep CNN on test set of SaR dog search and rescue dataset.

Furthermore, Figure 13 shows the confusion matrix for the bark and nonbark classes of the lightweight CNN model. Evidently, the model produced on average more false negatives (2.1 bark activity examples were classified and not bark) than false positives (0.4 not bark activity examples were classified and bark) probably due to the fact that the dataset is imbalanced, containing significantly more nonbarking examples ( 6/1 ratio).

Apart from the performance metrics, since we were interested in deploying the selected DL model on the wearable device, we measured the inference time of the models. Table 8 presents the response times for both DL models measured on (a) a workstation equipped with an Intel(R) Core(TM) i7-7700K CPU (4 cores) running on max turbo frequency equal to 4.20 GHz and (b) a Raspberry Pi 4 computing module (quad-core ARM Cortex-A72 processor). We converted the developed models to a TensorFlow Lite format. TensorFlow Lite is a set of tools that enables on-device machine learning such as mobile, embedded, and IoT devices. As a result, the TensorFlow models were converted in a special efficient portable format known as FlatBuffers (identified by the .tflite file extension), providing several advantages over TensorFlow’s protocol buffer model format (identified by the .pb file extension) such as reduced size and faster inference time. The performance of the models was not decreased after the conversion to .tflite format.

For our measurement purposes, we ran the models 10,000 times and then computed the average inferencing time. The first inferencing run, which takes longer due to loading overheads, was discarded. As expected the inference time for the .tflite formats is significantly lower than those of the .pb formats. Moreover, since the objective was to deploy the model to a Raspberry Pi 4 we selected to use our Deep CNN. Even though it achieved a 0.32% lower F1-score, it is significantly faster (almost x7 times) than the Deep CNN TL model enabling real-time inference at the edge of the network.

## 6. Validation of the Proposed Implementation

The proposed system was validated in an abandoned and demolished hospital southwest of Madrid, running two scenarios with the assistance of different SaR dogs. Similarly to the data collection process, a first responder had the role of the “victim”, and was hidden somewhere in the arena among the ruins. Then, a SaR dog with the developed wearable mounted on its back started its SaR mission.

During this process we measured the accuracy and F1-scores of the developed bark detection and activity recognition models separately. Moreover, we estimated the overall F1-score for notifying the K9 handlers whether the victim was found or not. This is achieved by injecting an alert rule on the mobile that is triggered when the SaR dog is barking and standing simultaneously, which is what it is trained for denoting that it has found a missing person.

Table 9 presents the classes of the collected singals (IMU and audio). Not all of the motion signals were annotated. This is due to the fact that the SaR dog was missing (e.g., was behind the debris) or there was an overlap in the activities for a 2 s window (e.g., the SaR dog was searching for the first 800 ms and then stopped moving for the rest 1200 ms). Thus there are presented less examples than the total amount.

The obtained F1-scores and the corresponding accuracy results are presented in Table 10. The developed deep CNN activity recognition model achieved a F1-score equal to 91.21% and 91.26% accuracy, while the bark detection model acquired 99.08% F1-score and 99.77% accuracy. In particular, the latter provided only two false positives (i.e., the misclacified “not barking” as “barking”), and these, also, triggered the alert notification providing the same F1-score and accuracy metrics for the overall victim detection task.

**Figure 14:**
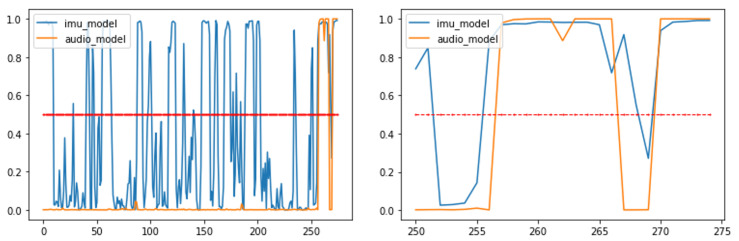
Plots displaying outputs of developed DL models during 275 s of 1st SaR scenario (**left**). Red line denotes threshold value (i.e., 0.5) for classifying an audio signal as “bark” and IMU signal as “stand”. The final 25 s of this scenario are displayed on (**right**) plot.

**Figure 15:**
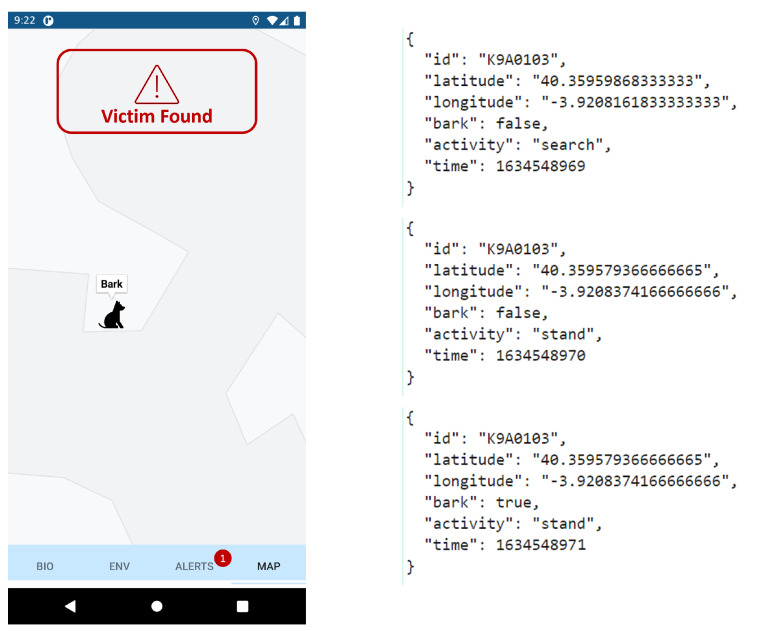
Mobile screenshot displaying generated alert in 1st SaR scenario (**left**), and corresponding KAFKA messages, with last one triggering alert rule (**right**).

Moreover, the developed solution was able to operate in real-time on the field, exploiting data processing at the edge, and it enabled the first responders to be aware of the K9 position and its behavior. Figure 14 and Figure 15 display a summary plot of the outputs of the DL models and the received smartphone notifications with respect to the received KAFKA messages, respectively. A video displaying these results and the whole validation procedure can be found here (https://www.youtube.com/watch?v=704AV4mNfRA, accessed on 20 January 2022).

## 7. Discussion

One of the main advantages of the current work is that it exploits edge computing to process in real-time the generated data before transmitting them through the network. In particular, in the case where there is no Wi-Fi available and the RF module is not efficient to send streaming audio data since the maximum data rate is 250 kbits/s, and the necessary rate for a medium quality audio signal is equal to 192 kbit/s, let alone the need to transmit the IMU signal and the GPS coordinates. In addition to this, to expand the data transmission range we reduced the data rate to 10 kbit/s, making it impossible to transmit the produced raw signals.

Moreover, the inclusion of IMU sensors is significant since the included SaR dogs are trained to bark and stand still when identifying a missing/trapped person. Thus, it reduces the false positives (i.e., victim found recognition) in the case the algorithm outputs that the dog barks but it is not standing or the dog falsely produces a barking sound. Furthermore, micro movements where the dog is confused (e.g., makes small circles) or is sniffing are not noticeable (i.e., the displayed coordinates will indicate it as standing) though the GPS signal, due to its estimation error (could reach up to 5 m), but are classified as searching by our algorithm.

However, one limitation of the approach is the activity recognition algorithm’s performance. Even though the overall accuracy is high, having an average of 7.32 misclafications in 100 s time span, for a critical mission application where even a second matters, this is not considered to be low, mainly due to the fact that the provided algorithm has not “seen” the examples of the dogs included in the test set. In other words, the behavioral patterns of some dogs are not close to the others and having more training data would be beneficial for the algorithm’s performance [51], a case that will be explored in the future.

Another possible limitation is that of the activity recognition algorithm’s generalizability in different dog breeds and environments. The SaR dogs included in training and evaluation were German Shepherds, American Labrador Retrievers, Golden Retrievers, Belgian Malinois, or mixed breeds (of the aforementioned) and ranged from 20 kg to 32 kg dogs. Moreover, the training and evaluation environments (arenas) were relative small areas with a lot of obstacles, such as debris. Thus, the algorithms performance on bigger SaR dogs (e.g., Saint Bernard) and wide open areas was not tested (e.g., forest covered with snow).

Finally, the current work has followed the guidelines regarding the Ethics Code (https://escuelasalvamento.org/wp-content/uploads/2021/04/Codigo-Etico_vf.pdf, accessed on 5 August 2021) of K9 training and the participating SaR dogs did not undergo any extra training for the purposes of this paper.

## 8. Conclusions

In this paper, we proposed a novel implementation that performs dog activity recognition and bark detection in real-time to alert the dog handler (a) about the dog position and (b) whether it has found the victim during a search and rescue operation. The proposed solution can significantly aid the first aid responders in search and rescue missions, especially in places where the rescuers either are not possible to enter, e.g., below debris, or if they cannot have the rescue dog within their line of sight. To realize thins, the candidate implementation incorporates CNNs, which have the ability to extract features automatically, attaining the highest accuracy compared with other known ML algorithms. In particular, it attained an accuracy of more than 93% both in activity recognition and bark detection in the collected test datasets and managed in both discrete validation scenarios to classify and alert the rescuer at the time that the dog managed to find the victim.

## Figures and Tables

**Table 1:** Summary of related work on Dog Activity Recognition (DAR).

Ref.	Location	Sensors	No. of Activities	Subjects	Algorithms
[20]	back	3-axial accelerometer and gyroscope	-	2 dogs of 2 breeds	only measurements from the sensors were performed
[21]	collar	3-axial accelerometer	17	18 dogs of 13 breeds	k-NN
[22]	collar	3-axial accelerometer	8	51 dogs of mainly 8 breeds	analytical algorithms created by the developers
[23]	collar	Heart rate, galvanic skin resistance, and body temperature	-	various dogs	only measurements from the sensors were performed
[24]	collar	3-axial accelerometer	-	6 dogs	generalized linear mixed effect models fit to the activity
[25]	withers	3-axial accelerometer and gyroscope	7	24 dogs of 2 breeds	SVM
[26]	back	ECG, PPG, Inertial Measurement Unit (IMU)	various	5 dogs of 4 breeds	only measurements from the sensors were performed
[27]	collar, vest, and forelimb	3-axial accelerometer	various	4 dogs of 4 breeds	only measurements from the sensors were performed
[28]	collar, tail	3-axial accelerometer and gyroscope	7	10 dogs of 9 breeds	Random Forest, SVM, KNN, Naïve Bayes, ANN
[6]	back, collar	3-axial accelerometer and gyroscope	7	45 dogs of 26 breeds	Decision Tree, SVM
[29]	collar	3-axial accelerometer	15	more than 2500 dogs of multiple breeds	Deep learning classifier

**Table 2:** Summary of the existing SaR solutions based on animal wearables.

Ref.	Animal	Sensors/Actuators	Communic. with Rescuer	Edge Data Processing	Delivery Package	Communication with Victim	Search through Debris	Extra Animal Training	No Welfare Concerns	No Rescuer Guidance Needed
[2]	dog	GPS, Bite sensor	Yes	No	No	No	No	Yes	Yes	Yes
[42]	dog	GPS, Bite sensor, Proximity Sensor, Tug sensor	Yes	No	No	No	No	Yes	Yes	Yes
[7,43]	dog	Accelerometer, Gyroscope, Magnetometer	Yes	Yes	No	No	No	Yes	Yes	Yes
[44]	dog	GPS, Camera, Thermographic camera, Infrared camera, Microphone, Speaker, Light.	Yes	No	No	Yes	No	No	Yes	Yes
[45]	dog	GPS, Microphone, Speaker, Light.	Yes	No	No	Yes	No	No	Yes	Yes
[46]	dog and robot snake	Bark detector (RobotSnake: Microphone, Camera)	No	No	Yes	Nos	Yes	Yes	Yes	Yes
[47]	rat	Microstimulating electrodes, Pressure sensors	No	No	No	Nos	Yes	Yes	No	No
this work	dog	Accelerometer, Gyroscope, Microphone, Camera, Vibrator, Speaker	Yes	Yes	Yes	Yes	Yes	No	Yes	Yes

**Table 3:** Electrical characteristics of device.

Parameter	Minimum	Typical	Maximum	Unit
**Absolute Maximum Ratings**				
Vin—Input Voltage	0.3	-	28	V
Iin—Input Current	-	-	1.6	A
Ichg—Battery Fast Charge Current	0.895	1	1.105	A
Vbat—Battery Charge Voltage	4.16	4.2	4.23	V
Operating Temperature	−40	-	100	°C
Storage Temperature	−65	-	150	°C
**Recommended Operating Conditions**				
Vin—Input Voltage	4.35	5	6.4	V
Operating Temperature	−40	-	85	°C

**Table 4:** Number of samples for each search and rescue session for four monitored activities.

Session No.	Standing	Walking	Trotting	Running	Searching
1	32	0	38	0	38
2	44	0	28	0	28
3	37	4	20	0	24
4	22	23	80	23	126
5	18	0	10	0	10
6	47	38	27	4	69
7	34	0	13	0	13
Total (542)	234	65	216	27	308

**Table 5:** Description of selected features.

Feature	Description
Mean	Average value
Min	Minimum value
Max	Maximum value
Median	Median value
Standard deviation	Measure of dispersion
Skewness	The degree of asymmetry of the signal distribution
Kurtosis	The degree of peakedness of the signal distribution

**Table 6:** Per dog accuracy of each Machine Learning (ML) model on dog activity recognition dataset.

Accuracy (%)								
Method	Dog 1	Dog 2	Dog 3	Dog 4	Dog 5	Dog 6	Dog 7	Avg
LR	91.43	90.28	96.72	95.27	100.0	87.93	87.23	92.69
k-NN	94.28	93.06	100.0	89.86	100.0	85.34	73.34	90.70
DT	90.57	86.11	90.49	89.32	92.85	82.24	79.14	87.25
RF	91.71	91.67	98.69	94.86	100.0	85.17	87.23	92.76
Deep CNN	91.14	93.06	100.0	95.00	100.0	84.66	91.91	93.68

**Table 7:** Performance of developed ML models on SaR dog bark detection dataset.

Method Name	Accuracy	F1-Score
Deep CNN TL [49]	99.34%	98.73%
Ours Deep CNN	99.13%	98.41%

**Table 8:** Mean inference time measure in milliseconds for each model.

Model	Intel I7-7700K CPU	Raspberry Pi 4
Deep CNN TL [49] .pb format	175.64 ms	839.59 ms
Ours Deep CNN .pb format	39.1 ms	116.88 ms
Deep CNN TL [49] .tflite format	25.59 ms	170.10 ms
Ours Deep CNN .tflite format	5.84 ms	25.53 ms

**Table 9:** Number of samples for two SaR evaluation scenarios.

Session No.	Standing	Searching	Barking	Not Barking	Duration (Sec)
1	57	98	17	258	275
2	167	170	40	555	595
Total	224	268	57	813	870

**Table 10:** Obtained F1-score and accuracy results for tasks of activity recognition, bark detection, and victim found recognition regarding two evaluation SaR scenarios.

Task	Accuracy	F1-score
Activity recognition	91.26%	91.21%
Bark detection	99.77%	99.08%
Victim found recognition	99.77%	99.08%

## Abbreviations

The following abbreviations are used in this manuscript:

DL	Deep Learning
CNN	Convolutional Neural Network
SaR	Search and Rescue
IMU	Inertial Measurement Unit
SIM	Secure IoT Middleware
PCOP	Portable Commmand Center
SVM	Support Vector Machines
LR	Logistic Regression
k-NN	k-Nearest Neighbours
DT	Decision Tree
RF	Random Forest
ANN	Artificial Neural Network
AAR	Animal Activity Recognition
DAR	Dog Activity Recognition
